# AI-mediated environments and the reconfiguration of symbolic convergence: cognitive implications for shared meaning formation

**DOI:** 10.3389/fpsyg.2026.1859863

**Published:** 2026-06-02

**Authors:** Jun-Yi Chen, Ting-Yun Xu, Tun-Yuan Tien

**Affiliations:** 1 Fuzhou University of International Studies and Trade, Fuzhou, China; 2 Research Center for the Inheritance and Innovation of Handicraft Culture of Humanities and Social Science Base of Fujian Province Universities, Fuzhou, China; 3 Xiamen University of Technology, Xiamen, China; 4 Xiamen University, Xiamen, China

**Keywords:** algorithmic mediation, artificial intelligence, cognition, human-AI interaction, interpretation, shared meaning, social meaning, symbolic convergence

## Abstract

Artificial intelligence is increasingly embedded in everyday communication, not only as a tool for generating or modifying messages, but as a condition that reshapes how meanings become available, circulated, and stabilized. Existing research has often focused on trust, anthropomorphism, or user attitudes toward AI. Less attention has been given to how AI-mediated environments may alter the process through which meanings become collectively shared. This article addresses that gap by using Symbolic Convergence Theory to examine how AI-mediated environments reshape the process through which meanings become shared, stabilized, and cognitively available. It treats fantasy themes, fantasy types, symbolic convergence, and rhetorical vision as linked levels in the formation of shared symbolic reality. Building on this framework, the article argues that AI-mediated environments reconfigure symbolic convergence in three ways: by accelerating the production of symbolic materials, expanding their circulation, and reinforcing their stabilization through repetition and prioritization. These changes matter not only at the level of communication, but also at the level of cognition. When meanings are increasingly encountered in prestructured, repeated, and personalized forms, interpretation may become narrower, more dependent on familiar cues, and more shaped by shifting cognitive authority. Rather than treating AI simply as an artificial social actor, the article proposes that AI should also be understood as part of a transformed symbolic environment within which human cognition operates. This perspective offers a conceptually grounded account of how AI reshapes shared meaning formation and, in doing so, alters the conditions under which human interpretation and judgment take shape.

## 1 Introduction

Artificial intelligence has become increasingly embedded in contemporary communication, not merely as a technical aid but as a mediating condition that reshapes how interaction is organized, how messages are interpreted, and how communicative contexts acquire meaning. In this sense, AI-mediated communication should not be reduced to a simple question of whether machines can imitate human expression. More fundamentally, AI now participates in the production, modification, and circulation of messages, thereby entering the symbolic environment within which human communication takes place (Hancock et al., 2020). Once AI is situated at this level, the issue is no longer confined to user attitudes toward technology, but extends to the broader conditions under which meaning is generated, shared, and stabilized.

Recent research has shown that AI-mediated environments are increasingly capable of structuring communication through mechanisms such as automated generation, adaptive response, personalization, and algorithmic filtering. These mechanisms influence what becomes visible, what is repeated, and what is rendered relevant within a communicative situation. Hancock et al. (2020) conceptualized AI-mediated communication precisely in these terms, arguing that AI can alter interpersonal communication by modifying or producing messages in ways that affect how interaction is experienced and understood. Empirical work has further demonstrated that AI-generated language can shape both task performance and interpersonal perception, suggesting that AI intervention does not simply assist communication but can alter the qualities of communication itself (Mieczkowski et al., 2021).

This transformation is especially important because communicative meaning does not arise from isolated messages alone. It emerges within contexts of salience, repetition, symbolic association, and interpretive orientation. AI-mediated environments increasingly participate in these conditions. Research on generative AI in everyday communication has suggested that AI systems are involved in redefining voice, cultural mediation, and everyday meaning production, thereby influencing how messages are framed, received, and situated within broader symbolic orders (Yin et al., 2025). Related discussions have also pointed out that AI-driven communication environments may privilege efficiency, accessibility, and personalization while simultaneously altering communicative authenticity, contextual depth, and interpretive nuance (Mirek-Rogowska et al., 2024; Shafik, 2026). Such findings indicate that AI is not external to communication, but increasingly constitutive of the conditions under which communication becomes meaningful.

At the same time, existing research has often focused on phenomena such as trust, anthropomorphism, user satisfaction, or interpersonal effects. Recent work on trust in AI has examined user trust, appropriate trust, trust calibration, trustworthiness, and the conditions under which AI-enabled systems become acceptable or reliable in human–AI interaction (Afroogh et al., 2024; Bach et al., 2024; Henrique and Santos, 2024; Mehrotra et al., 2024). A related body of research has addressed anthropomorphism in service robots, chatbots, AI-enabled technologies, and IT/IS environments, showing how humanlike cues, perceived agency, psychological distance, and system design shape users’ responses to AI systems (Blut et al., 2021; Li and Suh, 2022; Li and Sung, 2021; Wang and Ngai, 2026). Other studies have further connected anthropomorphic cues, chatbot service quality, and AI-based social support to interaction satisfaction, customer satisfaction, loyalty, self-disclosure, and interpersonal outcomes (Hsu and Lin, 2023; Lee and Lee, 2023; Merrill et al., 2025; Xie et al., 2023). These concerns are important, but they remain largely centered on users’ responses to AI rather than on the communicative process through which meanings become collectively shared. Even when recent studies address interactional change, they tend to emphasize altered user experience, pragmatic competence, emotional engagement, or relational effects, rather than the formation of shared symbolic realities across communicative environments (Eragamreddy, 2025; Fan and Li, 2026). As a result, an important conceptual gap remains: if AI-mediated systems reorganize visibility, repetition, communicative relevance, and interpretive context, then they may also be transforming the process by which meanings converge and become socially stabilized.

This article addresses that gap by shifting attention from attitudes toward AI to the changing conditions under which shared meanings are formed. Rather than asking whether AI appears human-like or whether users trust it, the present study asks how AI-mediated environments alter the conditions under which symbolic convergence occurs. This shift is necessary because AI is not only changing how messages are produced or circulated, but also reconfiguring the symbolic environment within which interpretation becomes possible. Seen from this perspective, AI is not simply a conversational object or informational assistant. It is an increasingly active condition of interaction that may reshape how meanings emerge, circulate, and solidify into shared interpretive orientations.

## 2 Symbolic convergence theory as a processual framework

For the purposes of this study, Symbolic Convergence Theory is most useful as a processual account of how shared symbolic reality emerges through interaction and interpretation. Its value lies not simply in offering a set of rhetorical terms, but in showing how meanings move from localized dramatization to wider collective uptake and, under certain conditions, to more stable forms of shared interpretation (Endres, 2016; Shields, 2008). Read in this way, the theory makes it possible to ask not only what symbolic materials circulate, but also how they become recognizable, repeatable, and capable of supporting broader interpretive alignment.

The relevance of this process can be further clarified by cognitive science research on shared reality. Shared reality refers to the perceived commonality of inner states, such as feelings, beliefs, and concerns about the world, with other people; it links social connection with the need to understand reality (Echterhoff et al., 2009; Higgins et al., 2021). This line of research also shows that communication is not only a channel for transmitting information, but a process through which interlocutors adjust messages, align understandings, and form shared representations of a communicative topic (Echterhoff and Schmalbach, 2018; Lau et al., 2001). In this sense, symbolic convergence can be read alongside shared reality research: both are concerned with how socially available meanings become cognitively and relationally consequential, although they approach this process from different disciplinary traditions.

Meaning-making and sensemaking research offers a further bridge between this communicative framework and the cognitive concerns of the present article. In psychology, meaning making has been treated as an interpretive process through which people relate particular experiences to broader systems of coherence, purpose, and significance (King and Hicks, 2021; Park, 2010). Wise-intervention research similarly emphasizes that people act on subjective meanings about themselves, others, and social situations, and that changes in these meanings can alter later judgment and behavior (Walton and Wilson, 2018). HCI and information-interaction research extend this concern to interaction with computational systems by treating meaning as part of how users engage with information and experience interaction as connected, purposeful, coherent, resonant, or significant (Mekler and Hornbæk, 2019; Ruthven, 2019). Related HCI work also examines how digital interfaces can recontextualize cultural materials toward multiperspectivity, inclusion, and sensemaking (Hirsch et al., 2024). In human-AI interaction, sensemaking becomes especially important because users must interpret outputs that may be useful yet difficult to understand; recent HCI work therefore examines explanations and sensemaking with AI, how explainability can support human-AI interaction, and the ways professionals contextualize and validate AI outputs in data-intensive settings (Kim et al., 2023; Luo et al., 2026; Vivacqua et al., 2019). These studies support the present article’s view that AI-mediated symbolic environments should be considered not only as channels of information, but also as conditions that shape how meanings are interpreted, made coherent, and treated as usable.

The process begins with fantasy themes. These are dramatized formulations through which participants symbolically organize people, motives, actions, and situations into communicable forms. Their importance lies in the fact that they render experience socially shareable. A fantasy theme is not merely an individual impression; it is a symbolic formulation capable of being recognized, repeated, and extended by others (Endres, 2016; Shields, 2008). This is why such themes often become consequential in contexts where groups are defining who they are, what kind of situation they face, or how a shared tension should be understood. Research on collective sensemaking and workplace identity shows that recurring dramatizations can become central to how participants articulate common experience and align themselves with one another (Gayatri, 2025; Gilmore and Kramer, 2019).

Fantasy types occupy a different place in the process. If fantasy themes are particular dramatized units, fantasy types are the more generalized patterns that make such units recognizable across situations. They are not separate from fantasy themes so much as abstractions formed through recurrence. Their significance lies in transferability: once a recognizable type is established, new themes can resonate more quickly because they echo an already familiar symbolic pattern. This helps explain why some meanings travel easily across texts, episodes, or communities. Ledbetter’s (2025) study of fan engagement illustrates this point well, showing how recurrent thematic forms can sustain symbolic involvement beyond any single message. Fantasy types therefore matter because they connect local dramatization to broader symbolic familiarity.

Symbolic convergence names the communicative movement through which these symbolic materials begin to align across participants. It is not best understood as one concept parallel to fantasy themes, fantasy types, or rhetorical vision. Rather, it refers to the overall process by which dramatized meanings are shared, elaborated, and made collectively resonant. Through this process, participants develop enough symbolic overlap to sustain shared emotions, interpretations, or motives, even when complete consensus is absent (Endres, 2016; Shields, 2008). Work on group decision making underscores the practical importance of this movement: dramatization can help participants coordinate judgment, maintain engagement, and orient themselves within a common frame of relevance (Horila, 2021). At the same time, symbolic convergence does not guarantee harmony. It may remain partial or unstable, and in some settings it may coexist with fragmentation or polarization, as shown in studies of algorithmically mediated public discourse (Saragih et al., 2026).

When convergence becomes sufficiently extensive and coherent, it may contribute to the formation of a rhetorical vision. A rhetorical vision is a broader symbolic framework through which participants interpret themselves, others, and the world they collectively inhabit. It emerges when multiple fantasy themes, often supported by recognizable fantasy types, become integrated into a more durable horizon of meaning (Endres, 2016; Shields, 2008). At this level, symbolic reality is no longer limited to local interactional uptake. It becomes capable of orienting identity, sustaining belonging, and legitimating action over time. Research on advocacy communities and collaborative settings shows both possibilities clearly: rhetorical visions can unify participants around a common symbolic world, but they can also compete or collide when different groups organize reality through incompatible frames (Aghazadeh, 2022; Broom and Avanzino, 2010).

What matters for the present article is the relation among these concepts. Fantasy themes provide the immediate dramatized substance of symbolic interpretation. Fantasy types make such themes more recognizable and portable across contexts. Symbolic convergence is the larger communicative process through which these materials gain collective traction. Rhetorical vision marks the broader interpretive framework that may emerge when convergence becomes sufficiently stabilized. Understood in this sequence, Symbolic Convergence Theory offers more than a vocabulary for describing discourse; it provides a layered model of how shared meanings are formed. This is precisely what makes it useful here. If AI-mediated environments alter what symbolic materials become visible, repeatable, and contextually salient, then they may also reshape the conditions under which this sequence unfolds.

## 3 AI-mediated environments as transformative conditions

To examine symbolic convergence in the age of AI, it is not enough to treat AI as a new communicative object. The more consequential shift lies in the fact that AI increasingly enters communication as an environmental condition. It shapes how messages are generated, circulated, selected, and encountered, and thus affects the symbolic circumstances within which meaning becomes available for uptake. This point is consistent with Hancock et al.’s (2020) definition of AI-mediated communication as interaction in which AI modifies, generates, or selects messages within human exchange. Once interaction is mediated in this way, the issue is no longer only what people say, but also how the environment shapes what becomes salient, repeatable, and contextually meaningful in the first place. For analytical clarity, AI-mediated environments should not be treated as a single mechanism. Generative AI mainly affects the production of symbolic materials, because it enables communicative content to be produced more quickly and at a larger scale (Biyela et al., 2024). Recommendation, ranking, personalization, and algorithmic filtering systems are more directly related to circulation, because they influence what content is selected, ordered, prioritized, or repeatedly shown within digital environments (Bojic, 2024; Kim and Sah, 2025). Predictive and assistive tools affect message formulation by shaping language use and making some response patterns easier to reproduce (Mieczkowski et al., 2021). These mechanisms may overlap in actual communication practices, but distinguishing them helps clarify how AI-mediated environments reshape symbolic convergence through different interventions in production, circulation, and stabilization (Biyela et al., 2024; Bojic, 2024; Mieczkowski et al., 2021).

Recent research suggests that one of the clearest consequences of this shift is a reorganization of symbolic salience. Algorithmic systems do not merely carry content; they participate in sorting, prioritizing, and legitimating it. Sánchez-Vera (2025) describes this as a form of critical algorithmic mediation, in which algorithms intervene in the circulation of knowledge and cultural meaning, thereby influencing which symbolic materials become more visible and authoritative. Similar concerns appear in work on intercultural narratives, where AI-driven semantic transfer may privilege dominant linguistic and cultural patterns while reducing contextual nuance (Chendeb, 2025). Visibility is therefore not simply a technical property of platforms. It also becomes part of the symbolic ordering of communication, because repeated exposure affects how messages are processed, remembered, and evaluated. Classic work on message repetition shows that repetition can alter cognitive responses, recall, and persuasive reactions to communication (Cacioppo and Petty, 1979). Research on the illusory truth effect similarly indicates that repetition can increase perceived truth by making statements feel more familiar or easier to process, even when repetition varies in wording or source (Fazio, 2020; Pillai and Fazio, 2025; Unkelbach and Rom, 2017). Communication research further suggests that repetition matters within dynamic framing and counterframing processes, where the timing and recurrence of frames can shape their effects across audiences (Chong and Druckman, 2013). At a broader disciplinary level, mass communication research has long treated media effects, exposure, information processing, and changing communication environments as central concerns, even as the field’s paradigm continues to shift (Lang, 2013). Applied communication research also emphasizes that public understanding is often supported by simple and clear messages that are repeated over time by trusted messengers (Maibach et al., 2023). In AI-mediated environments, what is repeatedly surfaced may therefore become easier to recognize, easier to recall, and easier to accept as meaningful, not because repetition guarantees agreement, but because it increases the cognitive availability and communicative familiarity of certain symbolic materials.

This matters even more when repetition is partly built into the communication environment itself. Markham (2024), writing from the perspective of symbolic interaction, shows that predictive systems such as auto-predict do not stand outside interaction but become woven into it by offering plausible continuations that shape conversational flow. Hohenstein et al. (2023) similarly demonstrate that AI-assisted communication affects both language use and social relationships. Their findings are important not because AI changes wording in a superficial sense, but because patterned suggestions and repeated linguistic forms can gradually normalize certain styles of response and interpretation. Under these conditions, repetition is no longer only the product of human emphasis or collective uptake. It can also be systemically produced, which gives some symbolic patterns a greater chance of becoming familiar, socially legible, and eventually taken for granted.

At the same time, AI-mediated environments influence how context is framed before interpretation even begins. Miarta et al. (2026) argue that algorithmic communication can reconfigure human agency by shifting interaction toward prompt-based and procedurally guided forms. In a related way, Kumar et al. (2025) note that AI-driven affective systems may standardize emotional expression, making some forms of feeling and response more communicable than others. What these studies suggest is that AI does not simply enter an already formed context; it increasingly helps define the terms on which context is recognized. It can narrow response possibilities, preformat communicative tone, and orient attention toward some meanings while marginalizing others. For the present study, this point is central. Symbolic convergence depends on the availability, recurrence, and intelligibility of symbolic materials within interaction. If AI-mediated environments are reorganizing those very conditions, then they are not peripheral to convergence. They are helping reshape the process through which shared meanings become possible.

## 4 Reconfiguring the process of symbolic convergence

The previous section argued that AI-mediated environments are not external to communication, but increasingly shape the conditions under which symbolic materials become available, noticeable, and repeatable. The task of the present section is to move one step further. If symbolic convergence depends on the circulation and uptake of dramatized meanings, then changes in communicative conditions must also change the process of convergence itself. The issue, then, is not whether AI creates an entirely new symbolic order, but how it changes the route through which fantasy themes gain traction, achieve wider recognition, and contribute to more stable rhetorical visions.

One of the clearest changes lies in the pace at which symbolic materials now enter communicative circulation. AI-assisted systems have made the production of text, image, and multimodal content more continuous and less dependent on the slower rhythms of human composition and exchange (Andriani, 2025; Geetha et al., 2024; Gonsalves et al., 2024). From the standpoint of symbolic convergence, this matters because fantasy themes do not emerge in isolation; they require symbolic material that can be noticed, repeated, and taken up by others. When content is generated more quickly and in greater volume, the field from which dramatized meanings may emerge becomes denser and more unstable at the same time. Themes no longer have to wait for extended interaction to become available. They can enter circulation rapidly, recur almost immediately in altered form, and begin accumulating symbolic familiarity before any sustained interpretive negotiation has taken place.

Speed alone, however, does not explain the shift. What changes under AI-mediated conditions is also the scale at which symbolic materials move. Systems of recommendation, personalization, and optimized dissemination do not simply distribute more content; they increase the likelihood that particular symbolic forms will travel farther, appear more often, and become recognizable across settings that are not tied to a single group or interactional context (Fang, 2024; Gombar, 2025; Raut et al., 2025). In earlier formulations of symbolic convergence, the development of shared meaning was often imagined through relatively bounded communities, teams, or publics. AI-mediated circulation complicates that picture. A fantasy theme may now achieve recognizability across dispersed audiences before it has been deeply worked through within any one of them. This does not eliminate convergence, but it changes its scale and texture. Shared symbolic references may form more quickly across wider networks, even when the interpretive depth supporting them remains uneven. This point requires a further conceptual qualification. Common visibility should not be equated directly with symbolic convergence, because repeated exposure in algorithmically mediated environments may stabilize symbolic familiarity without necessarily producing deeply negotiated shared meaning (Saragih et al., 2026). In digital and online contexts, symbolic convergence may remain partial, unstable, fragmented, or even antagonistic when platform visibility and narrative repetition reinforce competing rhetorical visions rather than a cohesive consensus (Saragih et al., 2026; Gyimóthy, 2013). This means that algorithmically repeated symbolic materials may become recognizable and affectively available before participants actively elaborate them through sustained interaction (Saragih et al., 2026). By contrast, stronger forms of symbolic convergence are more closely associated with participatory engagement, collective action, and the communicative constitution of shared meanings among interacting participants (Gyimóthy, 2013; Olufowote, 2006). The argument here is therefore not that AI-mediated repetition automatically produces full symbolic convergence; rather, such repetition may create a weaker precondition for convergence by making certain symbolic materials more visible, familiar, and easier to reactivate (Saragih et al., 2026). This qualification therefore does not claim that repeated algorithmic exposure is equivalent to full symbolic convergence; instead, it treats such exposure as a condition that may increase symbolic familiarity before deeper interactional negotiation takes place (Saragih et al., 2026; Olufowote, 2006).

These changes become most consequential when they affect stabilization. Symbolic convergence is not only a matter of exposure or spread. It becomes socially significant when some meanings begin to appear familiar, expected, and broadly intelligible. AI-mediated environments increasingly participate in that movement by repeatedly surfacing some symbolic materials while letting others recede. Hancock et al. (2020) define AI-mediated communication in terms of systems that modify, generate, or select messages within human interaction, and this selective role is crucial here. Once systems are involved in deciding what appears, what is reiterated, and what is made contextually prominent, repetition no longer belongs only to human communicative emphasis. It also becomes infrastructural. Mieczkowski et al. (2021) show that AI-mediated language affects interactional dynamics, while work on algorithm-driven discourse indicates that repeated exposure can reinforce patterned interpretation and reduce interpretive diversity (Gombar, 2025; Serttaş et al., 2025). Araújo’s (2025) formulation of the algorithm as a text is especially suggestive in this context, because it highlights that algorithms do not merely pass along meaning; they help organize the very conditions under which meaning becomes readable.

This is the point at which the implications for symbolic convergence become sharper. Fantasy themes are not simply more numerous in AI-mediated environments; some are more likely to become durable because they are repeatedly presented under conditions that favor recognition and recurrence. Fantasy types may likewise become more reusable, since familiar symbolic patterns can now be circulated and reactivated at a greater pace and across more settings. What is altered, then, is not only the beginning of convergence but also its cumulative force. The process becomes less dependent on prolonged social uptake alone and more entangled with patterned infrastructures of repetition, visibility, and prioritization.

Seen from this perspective, AI does not bypass symbolic convergence; it reorganizes the conditions under which convergence unfolds. The route from fantasy theme to rhetorical vision is still communicative, but it unfolds within an environment where symbolic availability is faster, circulation is wider, and stabilization is more systematically reinforced. This matters for the overall argument of the article because it identifies the specific point at which AI-mediated environments enter the process of shared meaning formation. The following section takes up the consequences of this shift more directly by asking what happens to interpretation and judgment when convergence is shaped under such conditions.

## 5 Cognitive implications of reconfigured convergence

The reconfiguration of symbolic convergence does not stop at the level of communicative process. Once symbolic materials are produced more quickly, circulated more widely, and stabilized through patterned repetition, they also begin to shape how users interpret what they encounter. The issue is therefore not only that AI-mediated environments contain more information or more symbolic cues. It is that these environments increasingly present meanings in forms that are already organized for recognition, uptake, and reuse. Under such conditions, interpretation becomes less a matter of open-ended sensemaking and more a matter of navigating meanings already structured for attention, familiarity, and acceptance.

One consequence is a shift in how users rely on interpretation itself. Research on AI dialogue systems suggests that repeated dependence on AI-generated responses can weaken critical engagement and encourage users to settle for available formulations rather than slower processes of reflection and evaluation (Zhai et al., 2024). This does not mean that users become passive in any simple sense, but it does indicate that mediated symbolic environments can make preformatted meanings more cognitively attractive. Related studies show that AI-assisted writing and AI-mediated language use affect how communication is perceived, including judgments of warmth, trust-building efficiency, and social connection (Mieczkowski et al., 2021; Purcell et al., 2025). What matters here is that these effects do not remain confined to isolated messages. When symbolic cues are repeatedly encountered in optimized or patterned forms, users may increasingly orient themselves through what is already familiar, available, and interactionally easy to process. The result is not the disappearance of interpretation, but a narrowing of the range within which interpretation is likely to move.

That narrowing becomes more visible when repetition and personalization begin to work together. Studies of algorithmic curation and echo chambers show that repeated exposure to prioritized content can reinforce certain frames while reducing encounters with alternative perspectives (Casapa and Puschmann, 2025; Gombar, 2025; Gombar and Boban, 2025). At the same time, psychological work on the truth effect suggests that familiarity itself can make information feel more acceptable or more likely to be true, even without additional evidential grounding (Stump et al., 2022). This is important for the present argument because reconfigured convergence does not merely increase exposure; it changes the symbolic conditions under which familiarity becomes meaningful. Personalization intensifies this pattern by aligning content more closely with prior preferences and recognizable cues, making interpretation feel more immediate while also reducing friction against prestructured meaning (Lee and Oh, 2012). In such environments, judgment is not only influenced by what users believe, but also by what they repeatedly encounter as already salient, already coherent, and already fitted to their symbolic expectations.

A further consequence concerns cognitive authority. AI-driven communication systems often occupy an ambiguous position in interpretation: they do not think in the human sense, yet their outputs may still be treated as usable guides for what matters, what counts as a reasonable answer, or what should be attended to next. Sun and Xu (2025) describe this in terms of hybrid cognitive authority and algorithmic subjectivity, pointing to the growing role of AI in shaping knowledge processes without possessing human cognition. Other studies suggest that this can affect users’ sense of autonomy and ownership over judgment, especially when decision support becomes difficult to question or when AI oversight changes how responsibility for error is experienced (Almog et al., 2024; Joers and De Luca, 2025). Even in contexts where AI support is beneficial, such as the expansion of analytical capacity or problem-solving assistance, the redistribution of interpretive labor remains significant (Olugboyega et al., 2026; Tsai et al., 2025). What is changing, then, is not simply the accuracy or usefulness of support, but the location of authority within the interpretive process itself.

At the same time, these cognitive consequences should not be understood as wholly deterministic. Critical AI literacy can help users recognize, question, and evaluate AI-generated or algorithmically organized outputs rather than treating them as neutral communicative conditions (Veldhuis et al., 2025). Studies of generative AI use also suggest that users may exercise agency by revising, post-editing, or giving feedback on AI-mediated language, even though such practices may require additional effort and may reduce communicative efficiency (Xiao et al., 2025). In educational and language-learning contexts, learner autonomy has likewise been linked to the ability to compare AI feedback, evaluate digital tools, and use ChatGPT-supported tasks in a more reflective manner (Lukešová and Jennings, 2025). From this perspective, human agency does not remove the structuring effects of AI-mediated environments, but it can introduce moments of friction, comparison, and redress within otherwise automated systems of symbolic production and circulation (Fanni et al., 2023; Veldhuis et al., 2025). The cognitive implications of AI-mediated convergence should therefore be understood as a tension between environmental structuring and reflective user agency, rather than as a one-way process in which users simply absorb preorganized meanings (Fanni et al., 2023; Xiao et al., 2025).

Taken together, these studies suggest that the cognitive implications of reconfigured convergence are best understood as changes in interpretive dependence, judgmental narrowing, and the distribution of cognitive authority. Users do not merely encounter more symbolic material under AI-mediated conditions; they increasingly encounter meanings that arrive preorganized for uptake, recurrence, and practical use. This is why the psychological consequence of AI-mediated convergence cannot be reduced to trust in technology or positive attitudes toward AI. The deeper issue is that understanding itself may begin to follow symbolic pathways that are more repetitive, more personalized, and less open to contestation than before. This suggests that AI confronts human cognition not only as an artificial interlocutor, but also as an environment that reorganizes the cues through which understanding ordinarily develops. In this way, the communicative transformation described in the previous sections reaches the level of cognition: AI-mediated environments shape not only how meanings circulate, but also how they come to feel intelligible, acceptable, and worth relying on.

## 6 Discussion

This article has argued that the significance of AI in everyday life lies not only in how people respond to artificial systems, but in how those systems reshape the conditions under which meanings become shareable and intelligible. A more basic issue is that AI-mediated environments increasingly shape the conditions under which meanings become noticeable, repeatable, and socially intelligible. Once this shift is taken seriously, AI is no longer only a communicative object or decision aid. It becomes part of the environment through which interpretation itself is organized.

From that starting point, the contribution of this article is relatively specific. It does not propose that AI replaces human meaning-making, nor does it claim that existing accounts of symbolic convergence are no longer useful. Rather, it shows that the process through which shared meanings take shape now unfolds under altered conditions. Fantasy themes still emerge, circulate, and sometimes stabilize into broader interpretive frameworks, but they do so in environments where symbolic materials can be produced more quickly, distributed more widely, and reinforced more systematically than before. What changes, then, is not the existence of convergence, but the way in which convergence becomes possible.

This point matters because the topic of AI and human cognition is not exhausted by questions about explicit attitudes toward artificial agents. Human understanding relies on patterned cues, recurring forms, and shared symbolic structures that help make situations legible. AI enters that level of experience by shaping what is available for recognition, what is repeated often enough to feel familiar, and what becomes easier to treat as relevant. In this sense, AI does not have to think like a person in order to affect how people think. It is enough that AI increasingly participates in the symbolic environment within which interpretation takes place.

Seen this way, the article speaks to the present topic through a more environmental account of cognitive change. The issue is not only whether evolved social-cognitive tendencies are directed toward AI as if it were human. It is also whether those tendencies now operate in environments where symbolic salience, recurrence, and uptake are partially organized by nonhuman systems. That shift suggests a form of mismatch at the level of interpretive conditions. Human cognition remains oriented toward making sense of socially available meanings, yet the conditions under which those meanings are selected, repeated, and stabilized are changing. AI therefore matters not only as an artificial partner, but as part of a transformed symbolic ecology.

Future empirical research could build on the distinctions developed in this article by examining different AI mechanisms separately. Studies of generative AI could analyze whether AI-produced texts, images, or multimodal materials introduce recurring symbolic themes into communication. Studies of recommendation, ranking, and personalization could examine how algorithmic systems affect the visibility and circulation of symbolic materials across audiences. Studies of predictive or assistive tools could investigate whether repeated language suggestions shape message formulation and response patterns over time. Future research could also distinguish among repeated symbolic exposure, weaker forms of symbolic familiarity, and more deeply negotiated forms of symbolic convergence.

A broader implication follows from this argument: research on AI and cognition may need to attend more carefully to the conditions under which meanings become collectively available before deliberate judgment is fully formed. This includes questions of repeated exposure, patterned familiarity, interpretive narrowing, and the changing location of cognitive authority. The value of the present article lies in bringing these issues into the same frame. By reconsidering symbolic convergence under AI-mediated conditions, this article shows how shared meaning formation itself becomes part of the cognitive challenge posed by artificial agents.

## Data Availability

The original contributions presented in the study are included in the article/supplementary material, further inquiries can be directed to the corresponding author.
